# Approximate Query on Temporal Knowledge Graphs via Two-Level Embeddings

**DOI:** 10.3390/e27121232

**Published:** 2025-12-05

**Authors:** Jiaxuan Liu, Xinyi Duan, Luyi Bai

**Affiliations:** 1 Sydney Smart Technology College, Northeastern University, Qinhuangdao 066004, China; 2 School of Computer and Communication Engineering, Northeastern University (Qinhuangdao), Qinhuangdao 066004, China

**Keywords:** approximate query, temporal knowledge graph, two-level embeddings

## Abstract

Approximate query on knowledge graphs (KGs) is an important and common task in real-world applications, where the goal is to return more results on KGs that match the query criteria. Previous approximate query methods have focused on static KGs. However, many KGs in real-world applications are dynamic and evolve over time. In this paper, we consider approximate queries in temporal knowledge graphs (TKGs) that may have specific timestamps in the predicates. We propose a Two-Level Approximate Query method (TLAQ) for temporal knowledge graphs based on the two-level embedding of vertex and graph. Specifically, we first improve the eigenmatrix of the GCN to enhance the embedding representation. On this basis, TLAQ defines relational reliability and attributive confidence at the vertex level. Then, we unify the encoding format of timestamps at the graph level to further strengthen the embedding model. Finally, we demonstrate the effectiveness of our proposed approach through a comprehensive experiment.

## 1. Introduction

**Figure 1:**
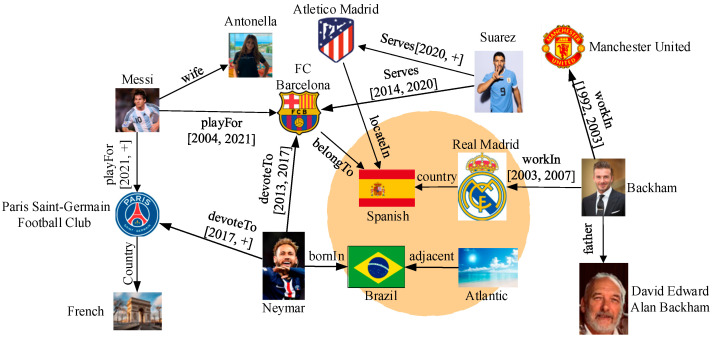
Example of temporal knowledge graph query instances showing entities, relations, and time-interval annotations.

Temporal knowledge graphs, such as DBpedia [1] and ICEWS [2], store information in the semi-structured form <h, r, t, time>, where h and t are the entity, r is the relation, and time is the timestamp held by r. Different entities or relations may have the same meaning, due to the flexibility of the semi-structured form. Disregarding the semantic similarity among annotated entities and relations may lead to the loss of approximate results that would otherwise meet the query constraints. Consider the examples shown in Figure 1. They show some samples of countries and celebrities. Assume that we need to answer the query “Please identify the FC Barcelona soccer players who have served since 2004”. If we ignore the query similarity, only the entity “Suarez” has a “serves” relationship with “FC Barcelona”. Obviously, Messi has also been playing for FC Barcelona between 2004 and 2021. Therefore, it is necessary to find a more comprehensive and complete answer by focusing on the similarity of entities and relations.

Some existing works focus on the importance of semantic similarity in queries. Zou et al. [3] model the query operations on TKG as graph transformations. They assigned different weights to each transformation to find multiple results. However, this method ignored the similarity between entities and did not consider the semantic information between predicates. Wang et al. [4] define path semantic similarity to find approximate predicates, but the time complexity of this method is relatively high. The FGqT-Match [5] algorithm is designed to reduce redundant computations by indexing subgraphs based on match-driven flow graphs. Meanwhile, the multi-label weight matrix was defined to evaluate approximate query graphs and reduce search space. There are also several TKG completion and forecasting methods [6,7]. The above research methods on approximation results are all based on the embedding model. However, if the query result is empty, simply using the embedding method alone may result in query failure. At this moment, relaxing the query criteria can produce the answer that is closest to the query intent.

To address the problem of empty results in the query, existing work reconstructs the original query into a new relaxed query by removing or relaxing the query criteria. These methods generate multiple approximate candidate query results based on four main models of similarity, rules, user preferences, and collaborative techniques. The similarity-based model [8] uses lexical analysis to find approximate results. The rule-based model [9] uses the semantics of query graph patterns and rewriting rules to perform relaxation. The model based on user preferences [10] relaxes overly constrained queries based on domain knowledge and user preferences. The model based on collaborative techniques [11] designs a new pruning strategy that greatly reduces the exponential search space for top-k relaxed queries. Recent advances in knowledge representation provide stronger foundations for semantic matching in query relaxation. For instance, HyCubE [12] introduces efficient three-dimensional circular convolutional embedding for knowledge hypergraphs, capturing complex structural patterns through hyper-relational modeling. The work by Ma et al. [13] incorporates historical trends with normalizing flow techniques to enable one-shot temporal reasoning. Further, Zhu et al. [14] propose multi-dimension rotations based on quaternion systems to effectively model diverse temporal patterns in knowledge graphs. All of these methods have been successful in relaxing the query to some extent. However, the approximate query generated by these methods may differ significantly from the user’s initial query, since these models cannot take into account expected answers that do not appear in the query results. Moreover, overly relaxed queries and irrelevant answers may produce query results that do not meet the user’s expectations.

Different from the existing approximate query methods, we propose an approximate semantic query method TLAQ for temporal knowledge graphs. This method divides the knowledge in TKG into relational triples and attribute triples. The specific embedding strategy for each of the two triples makes the continuous space vectors express the semantic information of entities or attributes more reasonably. In particular, the method solves the problem of empty results in the query. If the triples in the result graph are not found in the TKG, the method can still find an approximate result that satisfies the user’s initial query intent. The contributions of this paper are as follows:

- We define relational reliability and attributive confidence to find the approximate results at the vertex level and compare the semantic similarity at the graph level.
- We unify the encoding format of time information and encode predicate and time information jointly through LSTM.
- We conduct experiments on three real-world datasets to demonstrate the superior performance of the proposed approach.

The rest of this paper is organized as follows. Section 2 describes the related work of this paper. Section 3 gives the framework of our approach. Section 4 gives extensive experimental results, and Section 5 presents the conclusions and future work.

## 2. Related Work

### 2.1. Temporal Knowledge Graph Embedding

A wide range of A models exploit temporal dependencies to achieve improved performance. Early work such as Reference [15] extended static KG models (e.g., Tucker, DistMult) to temporal settings through four-dimensional tensor representations (<subject, predicate, object, time>), enabling episodic memory modeling in TKGs and inspiring subsequent research. The HyTE [16] model divides the TKG into different hyperplanes according to temporal information and maps entities and relationships to hyperplanes. Goel et al. [17] put forward DE series models to embed entities into time information on the basis of the static embedding method. They use CNN to learn the time perception representation of relations and use the DistMult scoring function to evaluate similarity. TA-DistMul1f [18] combines time and relationship into one dimension. The combined text sequence is calculated by LSTM to obtain the relationship with time characteristics. CyGNet [19] applies a replication mechanism to TKG for the first time. It combines replication and generation reasoning modes and refers to known facts from history when learning to reason future events.

The GCN, as an effective structural characterization method, captures structural dependencies between entities under the same timestamp through a message passing framework. On the basis of the R-GCN model, a cyclic event network RE-NET [20] model is proposed. This model is an autoregressive architecture for modeling the time series of multi-relational knowledge graphs. The RE-NET model further improves the semantic expression ability of TKG embedding. DySAT [21] pays attention to both graph structure and time evolution. Time-divided events learn neighborhoods’ information through self-attention and then learn triple similarity under time deduction through joint attention. EvoveGCN2 [22] obtains the structural features of a knowledge graph through the GCN and captures the evolution information of a knowledge graph through a cyclic neural network. Han et al. [23] put forward a link prediction model xERTE for future events. This model can query the related subgraphs of TKG and jointly model the graph structure and time context information.

### 2.2. Approximate Query Based on Embedding

In recent years, approximate query based on embedding has been extensively studied. Miller et al. [24] employs a memory table to store triples encoded into key–value pairs, which makes approximate query efficient. Sun et al. [25] propose a GNN model for multi-hop reasoning of heterogeneous graphs. PullNet [26] improves the graph query module by iteratively extending problem-specific subgraphs. Embedding KGQA [27] directly matches pretrained entity embedding with query embedding, which is computationally intensive. Bai et al. [28] divides graph embedding into vertex-level embedding and graph-level embedding. The model introduces an attention mechanism to learn graph-level embedding. In the graph query stage, vertex-level information is used to assist the similarity judgment of graph level. Yang et al. [29] propose Graph Path Networks (GPN), which combines the pretraining information based on an attention mechanism and cross-graph information. To address the challenge of sparsity in temporal knowledge graphs, Gao et al. [30] propose the Time-Weaver Query (TWQ) model, which leverages complex space embedding and exploits adjacent timestamp relations to reconstruct entity, relation, and timestamp embeddings.

Several methods have also been proposed to directly handle queries expressed in natural language. Chen et al. [31] put forward the HGNet model to solve the approximate query problem. The model gives the structure of a query graph and fills entities into the structure to complete the construction of a query graph. Liang et al. [32] decomposes a mapping query described in natural language into five subtasks of structured approximate queries. The method uses machine learning and a LSTM neural network model to learn the task automatically. Jin et al. [33] propose a question-answering system with relational constraints, which includes a dictionary construction module and a dictionary-based question-answering module. Zheng et al. [34] put forward a working framework to answer natural language questions in a user-interactive manner.

## 3. The Proposed Framework

### 3.1. Problem Formulation

**Definition 1.**  ***Temporal Knowledge Graph.** A temporal knowledge graph is defined as TKG = {E, A, T, RT, AT}, where E and A are the sets of entities and attributes, respectively.*

- *T is predicates set with temporal information. T = T_R_ × T_A_, where T_R_ is the relational predicate and T_A_ is the attribute predicate.*
- *RT = E × TR × E denotes the set of relation triples. <e_h_, t_r_, e_t_>* *∈ RT denotes a relation triple, where e_h_ is called head entity, e_t_ is called tail entity and t_r_ is called relational predicate.*
- *AT = E × T_A_ × A is the set of attribute triples. <e, t_a_, a>* *∈ AT denotes an attribute triple, where e is an entity, a is an attribute, and t_a_ is an attribute predicate.*

**Definition 2.**  ***Temporal Query Graph.*** *A temporal query graph is defined as TQG = {E_Q_* *∪ E_v_, A_Q_* *∪ A_v_, T_Q_, RT_Q_, AT_Q_}, where E_Q_* *⊆ E, E_v_ is a set of entity variables. A_Q_* *⊆ A, A_v_ is a set of attribute variables.*

- *T_Q_* *⊆* *T is the predicates set. Each variable, e_v_* *∈* *E_v_ or a_v_* *∈* *A_v_, is distinguished by a leading question mark symbol, e.g., ?e_v_ or ?a_v_. In particular, predicates are all in T, and there are no variables.*
- *RT_Q_ = (E_Q_* *∪* *E_v_) × T_R_ × (E_Q_* *∪* *E_v_). For each triple, an entity or attribute cannot be variable at the same time. <e_h_, t_r_, ?e_v_>* *∈* *RT_Q_ is called an entity query and ?e_v_ is defined as the target entity.*
- *AT_Q_ = (E_Q_* *∪* *E_v_) × T_R_ × (A_Q_* *∪* *A_v_). <e, t_a_, ?a_v_>* *∈* *AT_Q_ is called a relational query and ?a_v_ is defined as the target attribute.*

**Definition 3.**  ***Entity query and Attribute Query.*** *A entity query is defined as E_Q_ = {<e_h_, t_r_, ?e_v_>* *∈ RT_Q_ or <?e_v_, t_r_, e_t_>* *∈ RT_Q_}, where ?e_v_ is named as the target entity. An attribute query is defined as A_Q_ = {<e, t_a_, ?a_v_>* *∈ AT_Q_}, where ?a_v_ is named as the target attribute. While entity and attribute queries resemble traditional link prediction tasks, they differ in two key aspects:*

- *They are embedded in a temporal query graph structure that supports variable binding and time-aware matching;*
- *They are designed as components of more complex queries, enabling multi-hop reasoning and uncertainty handling via approximate matching.*

**Definition 4.**  ***Temporal result graph.*** *A temporal result graph is defined as TRG = {E_R_, A_R_, T_R_, RT_R_, AT_R_}, where E_R_* *⊆ E, A_R_* *⊆ A, T_R_* *⊆ T, RT_R_, and AT_R_ are relational triples and attribute triples that may not be in RT and AT.*

**Definition 5.**  ***Approximate query.*** *Consider a temporal knowledge graph G and a temporal query graph Q. Define a query procedure as an approximate query whose answer is the set of results that match G and Q, or the set of results that are closest to Q (when the matching result is the empty set). Approximate queries include entity query and attribute query in Definition 3.*

### 3.2. Overview

**Figure 2:**
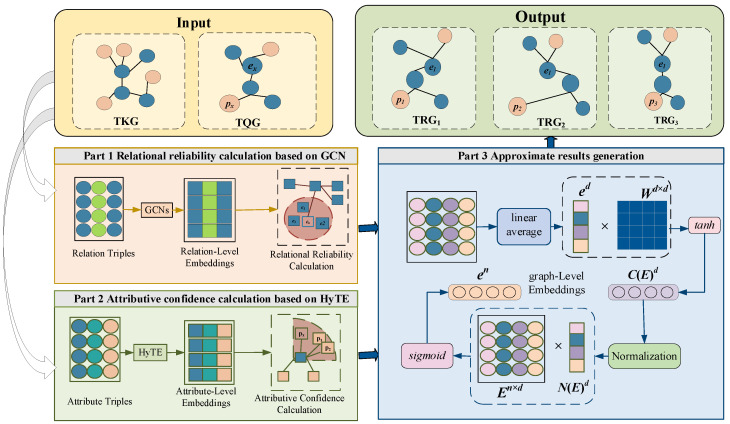
Framework of our approach.

This subsection proposes an approximate querying method on TKG based on two-level embedding. At vertex-level embedding, we define relational reliability and attributive confidence to find approximate entities and attributes. At graph-level embedding, time information is used to enhance the embedding representation and find the approximate results. The framework is shown in Figure 2.

**Part 1 Relational reliability calculation based on GCN.** For the relation triples, the GCN model is used to learn the embedding representation of the entity at the vertex level. Each vertex in the relation triples is transformed into a vector by combining the structural and semantic information of the vertices.
**Part 2 Attributive confidence calculation based on HyTE.** For the attribute triples, the translation distance-based representation model HyTE is used to obtain the embedding representations of vertices and edges in the attribute triple.
**Part 3 Approximate results generation.** To deal with the time information carried by predicates, a unified time coding format is defined to enhance embedded representation. After obtaining the embedded representation with time information, multiple triples will be weighted and averaged to obtain an embedded vector at the graph level. The distance between vectors will be used to measure the similarity among graphs.

### 3.3. Relational Reliability Calculation Based on GCN

Simple distance metrics fail in TKG approximation because they treat all dimensional proximities equally and cannot inherently account for structural integrity or temporal fitness, often leading to misleading high similarity rankings for structurally weak or temporally irrelevant candidates.

**Figure 3:**
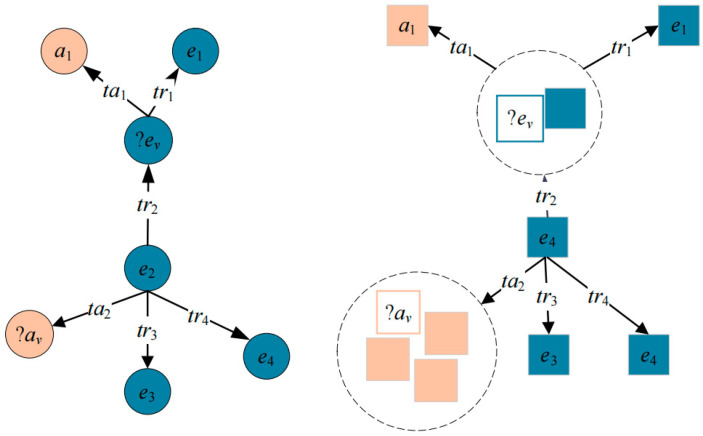
Examples of query tasks.

Figure 3 provides a detailed task description. On the left side of the figure is the representation before embedding the temporal query graph, which includes two types of queries: attribute queries <*e*_2_, *ta*_2_, ?*aᵥ*> and entity queries <?*e*_1_, *tr*_1_, *e*_1_>, <*e*_2_, *r*_2_, ?*eᵥ*>. On the right side of Figure 3 is the query graph after embedding. Although the temporal knowledge graph does not have results that can be directly returned, there are many candidate entities or attributes surrounding the target attribute or target entity, which are circled with dashed circles in the figure. These candidate entities or attributes have certain semantic similarities with the target entity or attribute and can be returned as results.

**Figure 4:**
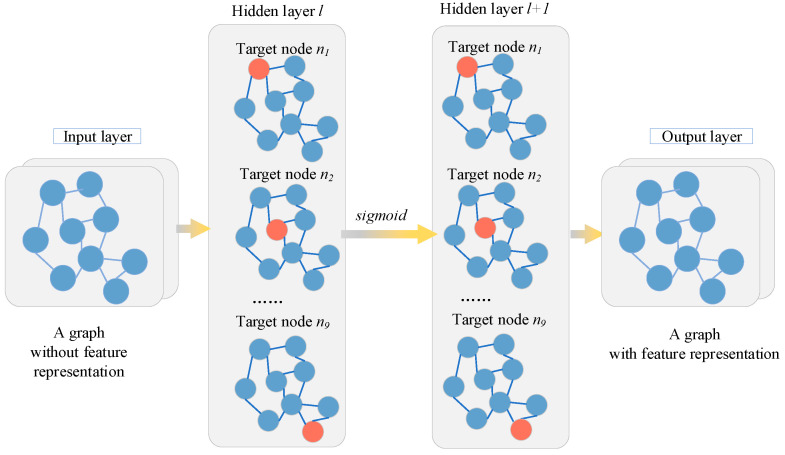
GCN structure based on modified feature matrix.

The Graph Convolutional Network has been proven to be effective in capturing information from graphs. It can learn the features of the pair of neighbor nodes, *e_h_* and *e_t_*, in the relational triple <*e_h_*, *t_r_*, *e_t_*>. The structure of the GCN is shown in Figure 4. The GCN takes the embedding representation $H^{\left(l\right)}$ of the nodes at layer *l* as input and computes the embedding representation at layer *l* + 1 by aggregating the embedding representations of neighbor nodes and its own nodes at layer *l*. The calculation method is as follows:

(1) $$H^{\left(l+1\right)}=\sigma (\tilde{A}H^{\left(l\right)}W_{0}^{\left(l\right)}+H^{\left(l\right)}W_{1}^{\left(l\right)})$$

(2) $$\tilde{A}=\;{\hat{D}}^{-\frac{1}{2}}A{\hat{D}}^{-\frac{1}{2}}$$

where σ is the activation function, and $\tilde{A}$ is the degree matrix as self-connected. A is the degree matrix. $W_{0}^{\left(l\right)}$ and $W_{1}^{\left(l\right)}$ are the learnable matrices. $\hat{D}$ is the diagonal node degree matrix of $\tilde{A}$.

A traditional GCN is limited to simple undirected networks and is therefore inadequate for modeling TKGs that involve multiple types of relationships between node pairs. For entity *a*, entity *b*, and entity *c*, if there are more edges (i.e., relationships) between entity *a* and entity *b* than between entity *a* and entity *c*, then the embedding representation of entity *a* should be closer to the embedding representation of entity *b* than the embedding representation of entity *c* in a continuous vector space. To better represent this difference, *A_ij_* is defined with the following equation:

(3) $$A_{\mathrm{ij}}(e_{i},e_{j},t_{r})\;=\;\sum_{<e_{i},\;t_{r},\;r_{j}>\;\in \;\mathrm{RT}}\mathrm{da}(e_{i},e_{j})\;\times \;\mathrm{re}(e_{i},e_{j},t_{r})$$

(4) $$\mathrm{de}(e_{i},e_{j})=\frac{{\mathrm{tail}}_{j}}{{\mathrm{head}}_{i}}$$

where *head_i_* is the number of relational triples with *e_i_* as the head node in the TKG, *tail_j_* is the number of relational triples with *e_j_* as the tail node in these triples, and *d_e_* can delineate the direction of relationships between entities.

The direction of relationship and the number of relationships between entities can reflect the importance of relationships. We define *re* to measure the importance of specific relationships. The formula is as follows:

(5) $$\mathrm{re}(e_{i},e_{j},t_{r})\;=\;\frac{\mathrm{nodes}\_i\;+\;\mathrm{nodes}\_j}{\mathrm{nums}\_r}$$

where *nums_r* is the number of triples of relational triples of the TKG with relation predicate *t_r_*, *node_i* is the number of these triples that contain node *e_i_*, and *node_j* is the number of these triples that contain node *e_j_*. *re* gives the weight of the relation.

For entity query, the embedding representation of the target entity needs to be calculated first. The embedding of the target entity obtained by GCN is called preliminary embedding $\tilde{e_{v}}$. In a query graph, if a target entity involves multiple relational triples, these triples need to be considered jointly when calculating the embedded representation of the target entity. Different triples have different effects on the embedded representation of target entities.

**Figure 5:**
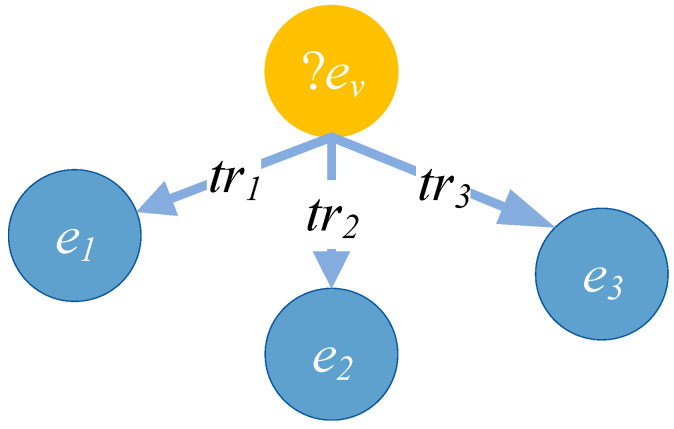
Entity query. An example of a temporal entity query represented as a query graph. The yellow node ?*ev* denotes an unknown entity to be inferred, while blue nodes *e*_1_, *e*_2_, and *e*_3_ are known entities. Directed edges labeled with relational predicates *tr*_1_, *tr*_2_, and *tr*_3_ represent constraints on the relationships between the target entity and known entities. This query seeks to find an entity that satisfies all three relation constraints simultaneously, possibly under temporal conditions (e.g., during a certain time interval).

An entity query is shown in Figure 5. The entity includes the relation triples <?*e_v_*, *tr*_1_, *e*_1_>, <?*e_v_*, *tr*_2_, *e*_2_>, and <?*e_v_*, *tr*_3_, *e*_3_>. When calculating the embedded representation of the target entity *e_v_*, <?*e_v_*, *tr*_1_, *e*_1_> contains more specific information compared to <?*e_v_*, *tr*_2_, *e*_2_> and <?*e_v_*, *tr*_3_, *e*_3_>. The relational triple <?*e_v_*, *tr*_1_, *e*_1_> deserves more attention. In this paper, the relationship triple influence factor *attr* is defined to measure the influence of a specific relationship triple on the target entity *e_v_*. The calculation method is given in Formula (6).

(6) $$\mathrm{attr}(e_{v},t_{r},e_{t})=\exp(-\frac{\sum_{<e_{v},t_{r},e_{t}>\;\in \;\mathrm{RT}}\left|\left\{e_{v}|<e_{v},t_{r},e_{t}>\;\in \;\mathrm{RT}\right\}\right|}{\sum_{<e_{v},t_{r}^{\prime },e_{t}^{\prime }>\;\in {\;\mathrm{RT}}_{Q}}\left|\left\{e_{v}|<e_{v},t_{r}^{\prime },e_{t}^{\prime }>\;\in \;\mathrm{RT}\right\}\right|})$$

The denominator of the index is the total number of all relational triples in the matching TKG, and the numerator is the number of specific relational triples containing *e_v_* in the matched temporal query graph. Based on the formula for *attr*, we can calculate the influence factors for the triples <?*e_v_*, *tr*_1_, *e*_1_>, <?*e_v_*, *tr*_2_, *e*_2_>, and <?*e_v_*, *tr*_3_, *e*_3_>.

In order to further calculate the embedding representation of the target entity *e_v_*, the final embedding of the target entity $\hat{e}$ is obtained by combining the preliminary embedding results and the influence factor *attr* of the triple. The formula is as follows:

(7) $$\hat{e_{v}}\;=\;\frac{\sum_{<e_{v},t_{r},e_{t}>\;\in \;{\mathrm{RT}}_{Q}}\mathrm{attr}(e,t_{r},e)\tilde{e_{v}}}{\sum_{<e_{v},t_{r}^{\prime },e_{t}^{\prime }>\;\in {\;\mathrm{RT}}_{Q}}\mathrm{attr}(e,t_{r}^{\prime },e_{t}^{\prime })}$$

where $\tilde{e_{v}}$ is the preliminary embedding and *attr* is the score of specific triples which contain *e_v_*.

After obtaining the final embedding representation of the target entity, we define the relational reliability *RR* to measure the semantic similarity between the two entities. This function maps the Euclidean distance into a value in [0, 1], where higher values indicate greater semantic similarity. We use this score to rank candidate entities and select the top-*k* most similar ones for approximate matching. The formula is as follows:

(8) $$\mathrm{RR}(e_{v},e)\;=\;\sqrt{\sum_{i=1}^{d}{\left(e_{\mathrm{vi}}\;-\;e_{i}\right)}^{2}}$$

where *e_v_* represents the target entity in the temporal query graph, *e* represents the approximate entity in the TKG, and *d* is the dimension of the embedded vector.

Relational reliability is used to find *k* entities that are closest to the target entity. For the *k* entities found, they correspond to *k* new triples <*e_k_*, *t_r_*, *e_t_*>, which are reliable relations of <?*e_v_*, *t_r_*, *e_t_*>. *e_k_* is called an approximate entity, which can replace *e_v_* as the approximate result. Algorithm 1 is as follows:

Breadth-first traversal is carried out first for each relational triplet containing the target entity. All neighbor entities of the target entity are added to *E* (lines 01–03). Then, the preliminary embedded representation of the target entity is calculated according to the GCN (line 04). For each approximate neighbor entity in *E*, its *attr* is calculated (lines 05–07). Based on the *attr*, the final embedded representation of the target entity is calculated (line 08). For the relational triples in the TKG, the similarity between the approximate entities in the triples and the target entities is calculated (lines 09–11). Finally, the approximate entities are returned by sorting by similarity (lines 12–13).
**Algorithm 1.** Approximate entities sorted based on relational reliability**Input**: temporal knowledge graph TKG, temporal query graph TQG **Output**: entity sorted result set *RT_R_*01**for** each *e_v_* **in** <*e_h_*, *t_r_*, ?*e_v_*> or <?*e_v_*, *t_r_*, *e_t_*>∈*EQ*02  *E* ← BFS (*e_v_*)//*e_v_* the root of breadth traversal search03**end for**04calculate the preliminary embedded representation of *e_v_*05**for** each *e_i_* **in** *E*06  calculate the *attr* of the relational triple corresponding to *e_i_*07**end for**08calculate the final embedded representation of *e_v_*09**for** each *e*′ **in** < *e*′, *t_r_*, *e_v_*> ∈ TKG10  calculate the similarity between *e*′ and *e_v_*11**end for**12according to the similarity, sort entities and save corresponding triple in *RT_R_*13**return** *RT_R_*

### 3.4. Attributive Confidence Calculation Based on HyTE

GCN is effective in learning the features of nodes with the same type in TKGs. It is insufficient for different types of nodes. For the attribute triple <*e*, *t_a_*, *a*> in the TKG, entity nodes and attribute nodes are different types. Most attribute triples in TKG are independent and do not contain complex relationship networks. When learning the embedding representation of attribute triples, the embedding method based on translation can deal with these relationships.

According to the translation-based embedding method, embed the attribute triples into a vector space. The preliminary embedding $\tilde{a}$ of attribute triples is calculated as follows:

(9) $$\tilde{a}\;=\;e\;+\;t_{a}$$

where $\tilde{a}$ is attribute, *e* is the entity, and $t_{a}$ is the attribute predicate.

In vector spaces of attribute triples, adjacent or similar entities share the same semantic information. The same entity always has different attributes, which interferes with attribute query. To remove the disturbance of irrelevant attribute predicates, we project adjacent entities onto the hyperplane corresponding to the attribute predicates in the attribute query and obtain the embedded representation of adjacent entities in the hyperplane of *t_a_*. The formula is as follows:

(10) $$e_{i}\;={\;e}_{i}\;-{\;t}_{a}^{T}e_{i}t_{a}$$

where *e_i_* is an embedded representation of an adjacent entity of *e*; *t_a_* is an embedded representation of the attribute predicate *t_a_*.

**Figure 6:**
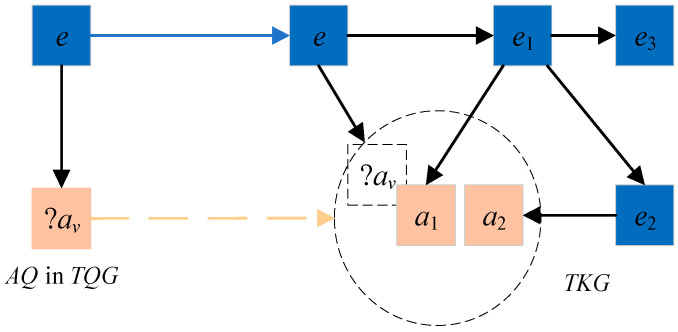
Attribute query. Illustration of attribute query processing when the target attribute ?*a_v_* is absent in the temporal knowledge graph, showing how the framework identifies contextually relevant approximate attributes (e.g., *a*_1_, *a*_2_) and their associated entities through embedding similarity scoring.

The attribute query <*e*, *t_a_*, ?*a_v_*> is shown in Figure 6. When this query is executed, there is neither an attribute predicate nor an attribute matching the attribute query <*e*, *t_a_*, ?*a_v_*> in the TKG. In the vector spaces of attribute triples, there are some approximate attributes (such as attribute *a*_1_ and attribute *a*_2_) that are similar to $\tilde{a}$ in TKG (represented by a square dashed box on the right of Figure 6). Moreover, most of the entities with these approximate attributes are adjacent to the entities in the attribute query (such as entity *e*_1_ and entity *e*_2_).

In order to further distinguish the distance between each adjacent entity and entity *e* in the attribute query, we define entity influence factor *atta* to measure the importance of adjacent entities to entity *e*.*atta*.

(11) $$\mathrm{atta}(e,e_{i})=\frac{\mathrm{sim}(e,e_{i})}{\sum_{<e_{i}^{\prime },\;t_{r},e>\in \mathrm{RT}⋁<e,t_{r},e_{i}^{\prime }>\in \mathrm{RT}}\mathrm{sim}(e,e_{i})}$$

The numerator represents the similarity between the adjacent entity *e_i_* and the entity *e* in the attribute query, and the denominator represents the sum of the similarity between the adjacent entities and *e*. The final embedding $\hat{a}$ of the target attribute is obtained by $\hat{a}$ and is calculated as follows:

(12) $$\hat{a}\;=\;\sum_{<e_{i}^{\prime },\;t_{r},e>\in \mathrm{RT}\;⋁\;<e,t_{r},e_{i}^{\prime }>\;\in \mathrm{RT}}\mathrm{atta}(e,e_{i})\tilde{a}$$

where $\tilde{a}$ is the preliminary embedding of the target attribute, and *atta* is the entity impact factor of the adjacent entity.

After obtaining the embedded representation of the target attribute, finding the attribute approximate with the target attribute in the TKG is needed. In order to measure the semantic similarity between the two attributes and find the attribute satisfying the query condition as much as possible, we define the attribute credibility *AC* to measure the likelihood that an observed attribute a in the TKG matches the target attribute $a_{v}$ in the query. The formula is as follows:

(13) $$\mathrm{AC}(a_{v},a)\;=\;\sum_{i=1}^{d}\left|a_{\mathrm{vi}}-a_{i}\right|$$

where *a_v_* represents the target attribute, *a* represents the approximate attribute, and *d* is the dimension of the embedded vector.

Attribute confidence is used to find *k* attributes that are closest to the target attribute. For the *k* attributes found, they correspond to *k* new triples <*e*, *t_a_*, *a_k_*> which are at attribute confidence values of <*e*, *t_a_*, ?*a_v_*>. *a_k_* is called an approximate attribute and can replace *a_v_* as the approximate result. Algorithm 2 is as follows:
**Algorithm 2.** Approximate attributes sorted based on attribute confidence**Input**: temporal knowledge graph TKG, temporal query graph TQG **Output**: attribute sorted result set *AT_R_*01**for** each *e* **in** <*e*, *t_a_*, ?*a_v_*> ∈ *AQ*02  *A* ← BFS (*e*)//*e* the root of breadth traversal search03**end for**04calculate the preliminary embedded representation of *a_v_*05**for** each *a_i_* **in** *A*06  calculate the *atta* of the attribute triple corresponding to *a_i_*07**end for**08calculate the final embedded representation of *a_v_*09**for** each *a*′ **in** <*e*, *t_a_*, *a*′>∈TKG10  calculate the similarity between a′ and *a_v_*11**end for**12according to the similarity, sort attributes and save corresponding triple in *AT_R_*13**return** *AT_R_*

Breadth-first traversal is carried out firstly for each attribute triplet containing the target attribute. All neighbor entities of *e* are added to *A* (lines 01–03). Then, the preliminary embedded representation of the target attribute is calculated according to the embedded model (line 04). For each entity in *A*, its *atta* is calculated (lines 05–07). Based on the *atta*, the final embedded representation of the target attribute is calculated (line 08). For the attribute triples in the TKG, the similarity between the approximate attributes in the triples and the target entities is calculated (lines 09–11). Finally, the approximate attributes are returned by sorting by similarity (lines 12–13).

### 3.5. Approximate Results Generation

**Figure 7:**
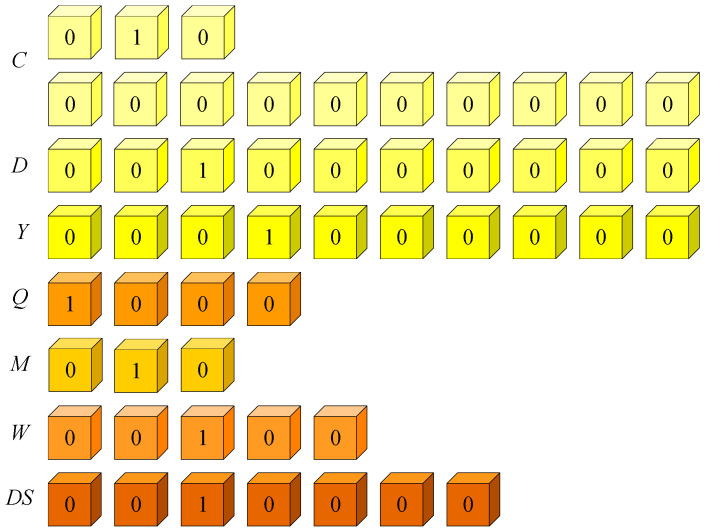
Encoding of time information in predicates.

Referring to the coding of time information in the literature [35], time information (*T*) is divided into seven levels, namely century, decade, year, quarter, month, week, and day. These seven time levels are represented by sub-vectors (*t*), which are centuries (*C*), decades (*D*), years (*Y*), quarters (*Q*), months (*M*), weeks (*W*), and days (*DS*). The first three vectors are used to represent the year, the *Q* and *M* vectors are used to represent the month, and the *W* and *DS* vectors are used to represent a specific day. Each dimension of the vector has a value of 0 or 1. The dimension of the *DS* vector is 7. The values of each sub-vector are given in Figure 7. The core reason we employ a seven-level time encoding is to achieve a comprehensive, multi-granularity representation of temporal information within temporal knowledge graphs (TKGs). Real-world events and facts possess varying durations and granularities, ranging from long-term facts spanning centuries to short-term events lasting only days or weeks. This seven-level structure allows the model to simultaneously capture the context from the broadest scale (century) down to the most precise date, thereby greatly enhancing both representational capacity and robustness.

Specifically, a century has a hundred years, a year has four quarters, a quarter has three months, a month has four weeks, and a week has seven days. The highest three digits of *C* represent the first 1000 years from *AD* 0, and the fourth-third digits represent the first hundred years in 1000 years. Number 1 indicates that in this time interval. The highest bit of *D* is indicated in the first decade, and 1 means that in this time interval. The highest bit of *Y* denotes the first year, and number 1 denotes that in this time interval. The highest digit of *Q* is the first quarter; number 1 is in this quarter. The highest digit of *M* is the first month of the quarter; number 1 is in this month. The highest bit of *W* represents the first week (assuming that the 1st of each month is the beginning of the first week and the week is seven days); number 1 means in this week. The highest bit of *DS* means the first day of a week; number 1 means in this day.

**Figure 8:**
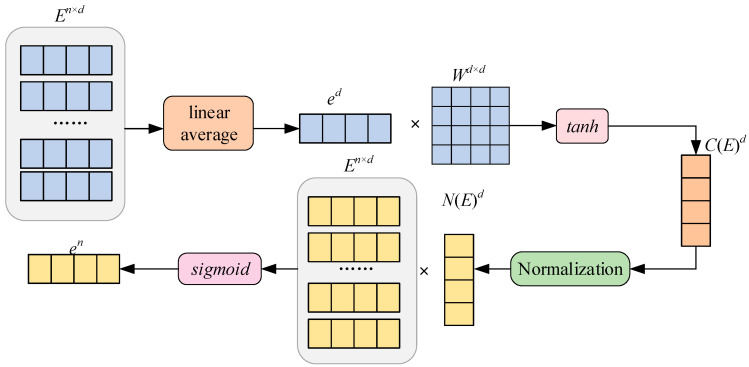
Embedding of graph level.

The encoding of time information and predicate vectors trained by Section 3.3 and Section 3.4 are put into LSTM. The predicate encoding with time information is obtained by using LSTM to jointly encode predicates and time. Section 3.3 and Section 3.4 judge the similarity between TQG and TKG at vertex level. In order to further calculate the similarity between the TQG graph and TKG, this section synthesizes the feature information of each vertex and edge from the perspective of embedding the whole graph and represents the whole graph with an embedding vector. The structure of the embedding model at the graph level is shown in Figure 8. It illustrates the graph-level embedding model, which functions as the final aggregation step in Part 3 of our framework. The model converts the set of vertex-level entity embeddings (*E^n^*^× *d*^) into a single, comprehensive graph embedding (*C*(*E*)*^d^*) by employing a **dual-branch structure**. The **top branch** calculates the **global information** of the graph through a **linear average** of the entity embeddings, followed by a weighted transformation and *tanh* activation to normalize the result. Simultaneously, the **bottom branch** incorporates the **importance or confidence** of individual entities (*E^n^*) using a $\text{sigmoid}$ function, which generates normalized weights to be applied to the entity embeddings before further processing. By combining the outputs of these two branches, the final graph embedding *C*(*E*)*^d^* is obtained, effectively encoding both the **global entity context** and the **local structural importance** into a fixed-size vector for subsequent similarity measurement.

In the embedding of the graph level, the input is the embedded representation *E* of the entity. The embedded representation of the entity is linearly averaged to obtain the global information *e^d^* of the entity first. After that, a nonlinear transformation of *e^d^* and *W^d^*^× *d*^ is conducted. The result is unified between [−1, 1] through the *tanh* activation function. The calculation formula is detailed in Formula (14):

(14) $$c(x)\;=\;\tanh\left(\left(\frac{1}{s}\sum_{k=1}^{s}x_{i}\right)W\right)$$

where *x_i_* is a concrete vector, and *W* is a learnable matrix.

In Formula (14), *c*(*x*) provides the structure information and characteristic information of the graph. In order to distinguish each entity in the graph, the specific weights of each entity are given. The attention function *c*(*x*) is the inner product with the embedded representation of each entity to obtain the improved entity representation, and the improved entity representation is linearly weighted to obtain the weighted embedded representation. The weighted embedding average of entities, predicates, and attributes is taken as the embedding representation of the graph and is computed as shown in (15):

(15) $$g\;=\;a(\sum_{i=i}^{n}f\left(e_{i}^{T}c\left(E\right)\right)e_{i}+\sum_{j=1}^{m}f\left(a_{j}^{T}c\left(A\right)\right)a_{j}+\sum_{k=1}^{b}f\left(r_{j}^{T}c\left(R\right)\right)r_{j})$$

where *a*() means averaging and *f*() is the activation function sigmoid, which guarantees that the vector is in the interval [0, 1].

After obtaining the embedded representation of the graph level, we define *sim* to calculate the similarity between the two graphs, and the calculation method is shown in Formula (16).

(16) $$\mathrm{sim}(g_{1},{\;g}_{2})=\sum_{i=1}^{d}\left|g_{1i}\;-{\;g}_{2i}\right|$$

## 4. Experiment

### 4.1. Experiment Setup

This paper uses the English version of DBpedia, which includes 6.7 M entities, 1.4 K relationships and 583 M triples. LC-QuAD is a commonly used question-and-answer dataset based on DBpedia. It contains 5000 queries and the standard results returned by queries. QALD, also based on DBpedia, is an open-data question-and-answer evaluation system that provides standard answers for each query. We chose QALD-6 and QALD-7 as verification sets. The analysis of the dataset is shown in Table 1.

All experiments are based on Windows 10 OS, Intel Core i5-1.9 GHz CPU, 8 GB RAM, and all algorithms are based on Python 3.7.

### 4.2. Experimental Results

**Figure 9:**
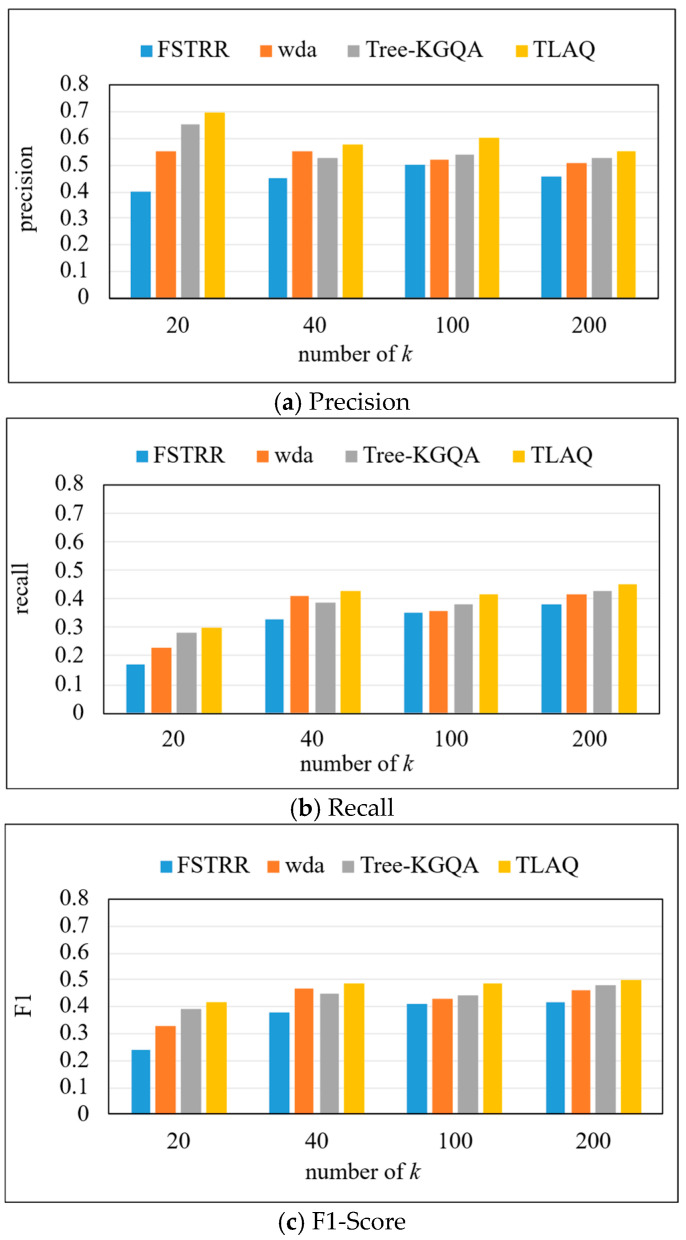
The precision, recall, and F1-Score on LC-QuAD.

We compare the TLAQ with FSTRR [36], WDA [37], and Tree-KGQA [38] in terms of precision, recall, and F1-Score to evaluate the effectiveness of our method. The precision, recall, and F1-Score on the LC-QuAD dataset are shown in Figure 9 and Table 2. Based on Figure 9, horizontal analysis shows that with the increase in k, the precision of the four algorithms has increased. Because most of the problems contained in this dataset are multi-result problems, the larger the *k* value, the more correct query results will be returned in the result set. Through longitudinal analysis, TLAQ shows advantages under different *k* values. When *k* = 20, compared with the FSTRR algorithm, the precision of TLAQ is improved by 75%. The average performance improvement rate of precision is 27.2% compared with the WDA algorithm.

This is because TLAQ processes entity query and attribute query separately and considers entity context information and attribute similarity information. The FSTRR algorithm only considers the weights of entities and attributes but does not consider their context information, which makes some semantically related entities lost in the query. Moreover, the FSTRR algorithm only considers the similarity of numeric attributes in query and does not include the semantic relevance of literal attributes in the screening of query conditions. It can also be found that the precision of TLAQ is less affected by *k*, which shows that TLAQ has certain stability and scalability. The F1-Score of all four algorithms increases as *k* increases, which indicates that the average performance of the algorithms improves as long as the number of allowed results is large enough. The F1-Score of TLAQ is relatively stable and it can reach about 0.42 even at *k* = 20.

**Figure 10:**
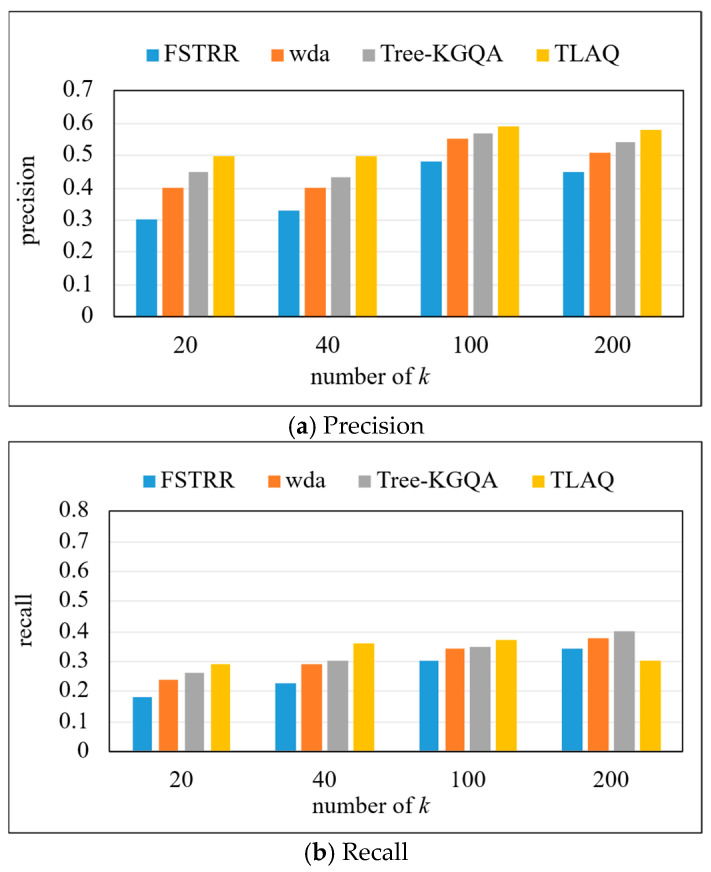

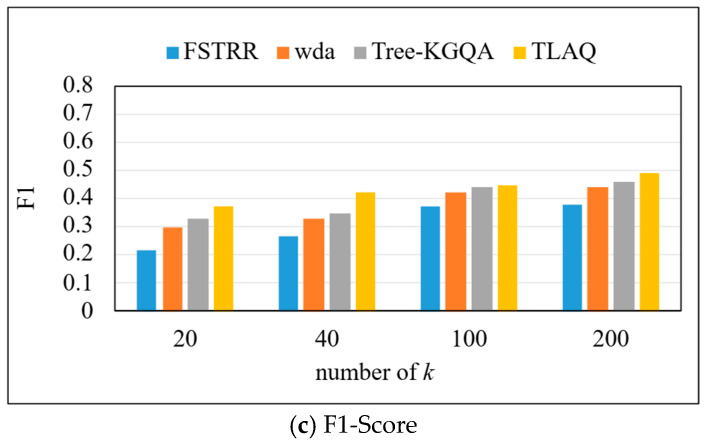
The precision, recall, and F1-Score on QALD-6.

The precision, recall, and F1-Score on the QALD-6 dataset are shown in Figure 10 and Table 3. The experiment selects the first 20, 40, 100, and 200 query results, respectively, to calculate the precision, recall, and F1-Score of each query problem and calculate their average. Based on the data in Figure 10, horizontal analysis shows that with the increase in k, the precision of the four algorithms increases. Under four different *k* values, TLAQ shows advantages. Compared with FSRTT, the average performance improvement rates of WDA and Tree-KGQA are 66.7%, 25% and 12.3%, respectively, at *k* = 20. Although the precision of TLAQ is improved compared with other algorithms, the overall performance of the model is not as good as that of LC-QuAD dataset. There may be two reasons for the above phenomenon. The first is that LC-QuAD is a question–answer dataset published in 2017. The questions in the dataset are relatively simple, including most single-hop problems and untyped questions. So, most algorithms can achieve 50% or even 60% accuracy on this dataset. The second reason is that the QALD-6 dataset contains richer questions with additional entity information in the query results. In addition, complex questions in QALD-6 account for more than 40% of the total questions, and these questions contain more complex syntactic structures and semantic relations than LC-QuAD.

Compared with the LC-QuAD dataset, the recall of the FSTRR algorithm decreases by 10.53%, the WDA algorithm decreases by 9.52%, and the Tree-KGQA algorithm decreases by 6.98% at *k* = 200. However, the TLAQ is relatively stable, and the F1-Score has been maintained at about 0.49. The F1-Score of the FSTRR algorithm decreased by 9.52%, the F1-Score of the WDA algorithm decreased by 4.35%, and the F1-Score of the Tree-KGQA algorithm decreased by 4.17%. However, the TLAQ is relatively stable, and the F1-Score has been maintained at about 0.49, with a fluctuation of 5.06%. This verifies the superiority of TLAQ from the side, and the performance on different datasets is not very different.

**Figure 11:**
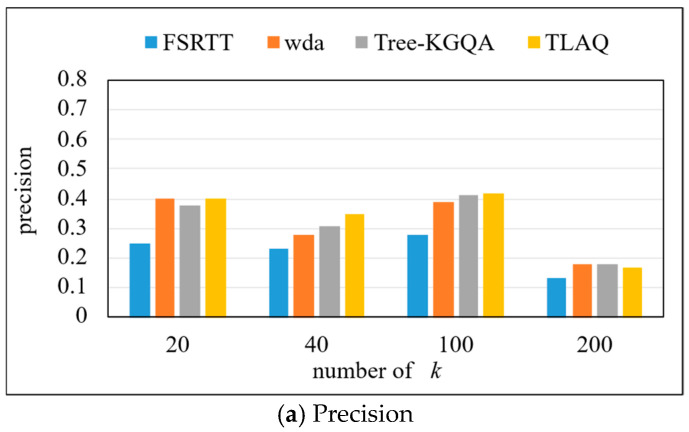

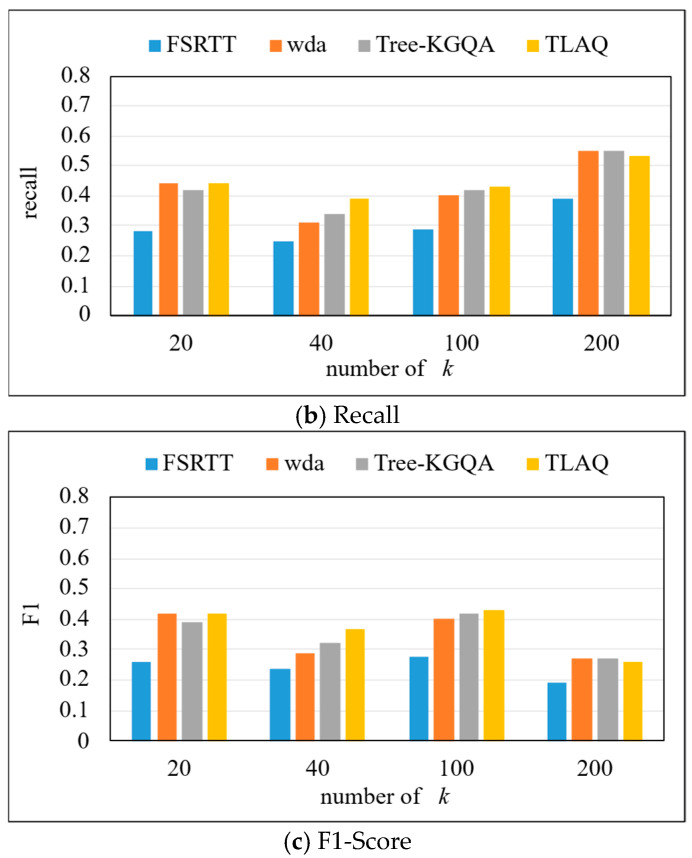
The precision, recall, and F1-Score on QALD-7.

The precision, recall, and F1-Score on the QALD-6 dataset are shown in Figure 11 and Table 4. The experiment selects the first 20, 40, 100, and 200 query results, respectively, to calculate the precision, recall, and F1-Score of each query problem and calculate their average. Based on the data in Figure 11, horizontal analysis shows that the precision of the four methods has the same change trend, which increases first and then decreases, and reaches its highest when *k* = 100. When *k* = 100, the precision of TLAQ reaches 0.59. TLAQ shows advantages under four different *k*. At *k* = 40, the average performance improvement rates of WDA and Tree-KGQA are 66.7%, 25%, and 12.3%, respectively.

These results can be attributed to TLAQ’s use of temporal information to enhance the embedded representation of entities and attributes. When answering questions related to time information, the calculated approximate entities are spatially close to the real entities. The WDA model only considers the number of question words contained in the entity labels and the edit distance between them when sorting approximate answers but does not weigh the entities. When the number of results is not limited, such as *k* = 200, the WDA model can return more correct results, which also leads to a negative average performance improvement rate of TLAQ when *k* = 200. However, in the actual query process, users often do not care about the results after ranking 100.

To study the execution efficiency of TLAQ, we analyzed its time complexity. In Algorithm 1, which ranks candidate entities based on relational reliability, the time complexity for finding candidate entities is O(S × Nₛ), where S is the number of target entities and Nₛ is the number of neighboring nodes. The time complexity for computing the triplet influence factor for each candidate entity is O(E), where E is the number of candidate entities. The time complexity for calculating the relationship between entities in each triplet and the target entity is O(S × N_1_), where N_1_ is the number of relational triplets in the temporal knowledge graph. Thus, the overall time complexity of Algorithm 1 is O(N((S × (N_1_ + Nₛ)) + E)). In Algorithm 2, which ranks candidate attributes based on attribute credibility, the time complexity for adding entities possessing the target attribute to the entity set is O(A × Nₐ), where A is the number of target attributes and Nₐ is the number of neighboring nodes of entities possessing the target attribute. The time complexity for computing the entity influence factor is O(E), where E is the number of candidate entities. The time complexity for calculating the relationship between attributes in attribute triplets and the target attribute is O(N_2_), where N_2_ is the number of triplets in the temporal knowledge graph. Hence, the overall time complexity of Algorithm 2 is O(N((A × (N_2_ + Nₐ)) + E)). The total time complexity of TLAQ is O(N((S × (N_1_ + Nₛ)) + A × (N_2_ + Nₐ)) + E)).

FSTRR compares each entity or attribute in the query with the corresponding entities or attributes in the triplets of the temporal knowledge graph one by one, with a time complexity of O(K × N), where K is the number of triplets corresponding to the query and N is the number of triplets in the temporal knowledge graph. wda mainly includes two algorithms: Algorithm 1 calculates the distance between vertices in the query graph and each vertex in the temporal knowledge graph, with a time complexity of O(N_1_ × N_2_ × N_3_), where N_1_ is the number of vertices in the query graph, N_2_ is the number of vertices in the temporal knowledge graph, and N_3_ is the number of edges satisfying the specified distance. Algorithm 2 generates query sentences and is a recursive algorithm with a time complexity of O(2^K^), where K is the number of results to be returned. Therefore, the total time complexity of wda is O(N((N_1_ × N_2_ × N_3_) + 2^K^)). Tree-KGQA decomposes the query graph into multiple trees, forming a forest, and finds answers by identifying K neighbors of nodes in the trees. The time complexity of this algorithm is O(F × R × K), where F is the number of triplets corresponding to the query graph, R is the number of entity relationships in the triplets, and K is the number of results to be returned.

Comparative analysis reveals that TLAQ does not involve recursive operations, and the rational use of pruning reduces the search space, resulting in relatively low time complexity.

## 5. Conclusions

In this work, we focused on approximate query problems of TKGs. We propose GCN-based relational reliability and translation distance-based attributive confidence in order to find approximate results at the vertex level. To address the temporal information of predicates, we unified the encoding format of temporal information and enhanced the embedding representation of predicates at the graph level. Finally, we introduced a TLAQ method to tackle the approximate query problem. The effectiveness of our approach has been validated through extensive experiments.

There remain several promising directions for future research. In particular, the applied bag-of-words embedding model can be further refined without compromising query efficiency. Additionally, we plan to extend our framework to handle complex logical query structures (e.g., conjunctions with temporal constraints) to better align with real-world query expressiveness requirements. This will bridge the gap between similarity-based approximation and practical query relaxation scenarios in temporal knowledge bases.

## Figures and Tables

**Table 1:** Statistics of the dataset.

Dataset	Query	Entity	Relationship	Temporal Information
LC-QuAD	5000	5042	615	Time point/Timestamp
QALD-6	450	383	378	Time point/Timestamp
QALD-7	215	265	313	Time point/Timestamp

**Table 2:** The precision, recall, and F1-Score on LC-QuAD (top-*k* = 20, 40, 100, 200).

Model	Precision				Recall				F1-Score			
	*k* = 20	*k* = 40	*k* = 100	*k* = 200	*k* = 20	*k* = 40	*k* = 100	*k* = 200	*k* = 20	*k* = 40	*k* = 100	*k* = 200
FSTRR	0.4	0.45	0.5	0.46	0.17	0.33	0.35	0.38	0.24	0.38	0.41	0.42
WDA	0.55	0.55	0.52	0.51	0.23	0.41	0.36	0.42	0.33	0.47	0.43	0.46
Tree-KGQA	0.65	0.525	0.54	0.525	0.28	0.39	0.38	0.43	0.39	0.45	0.44	0.48
TLAQ	0.7	0.575	0.6	0.55	0.3	0.43	0.42	0.45	0.42	0.49	0.49	0.5

**Table 3:** The precision, recall, and F1-Score on QALD-6 (top-k = 20, 40, 100, 200).

Model	Precision				Recall				F1-Score			
	*k* = 20	*k* = 40	*k* = 100	*k* = 200	*k* = 20	*k* = 40	*k* = 100	*k* = 200	*k* = 20	*k* = 40	*k* = 100	*k* = 200
FSTRR	0.3	0.33	0.48	0.45	0.18	0.23	0.3	0.34	0.22	0.27	0.37	0.38
WDA	0.4	0.4	0.55	0.51	0.24	0.29	0.34	0.38	0.3	0.33	0.42	0.44
Tree-KGQA	0.45	0.43	0.57	0.54	0.26	0.3	0.35	0.4	0.33	0.35	0.44	0.46
TLAQ	0.5	0.5	0.59	0.58	0.29	0.36	0.37	0.3	0.37	0.42	0.45	0.49

**Table 4:** The precision, recall, and F1-Score on QALD-7 (top-k = 20, 40, 100, 200).

Model	Precision				Recall				F1-Score			
	*k* = 20	*k* = 40	*k* = 100	*k* = 200	*k* = 20	*k* = 40	*k* = 100	*k* = 200	*k* = 20	*k* = 40	*k* = 100	*k* = 200
FSTRR	0.25	0.23	0.28	0.13	0.28	0.25	0.29	0.39	0.26	0.24	0.28	0.19
WDA	0.4	0.28	0.39	0.18	0.44	0.31	0.4	0.55	0.42	0.29	0.4	0.27
TreeKGQA	0.38	0.31	0.41	0.18	0.42	0.34	0.42	0.55	0.39	0.32	0.42	0.27
TLAQ	0.4	0.35	0.42	0.17	0.44	0.39	0.43	0.53	0.42	0.37	0.43	0.26

## Data Availability

Data will be made available on request.
